# Spike-in validation of an Illumina-specific variance-stabilizing transformation

**DOI:** 10.1186/1756-0500-1-18

**Published:** 2008-06-04

**Authors:** Mark J Dunning, Matthew E Ritchie, Nuno L Barbosa-Morais, Simon Tavaré, Andy G Lynch

**Affiliations:** 1 Department of Oncology, University of Cambridge, CRUK Cambridge Research Institute, Li Ka Shing Centre, Robinson Way, Cambridge, CB2 0RE, UK

## Abstract

**Background:**

Variance-stabilizing techniques have been used for some time in the analysis of gene expression microarray data. A new adaptation, the variance-stabilizing transformation (VST), has recently been developed to take advantage of the unique features of Illumina BeadArrays. VST has been shown to perform well in comparison with the widely-used approach of taking a log_2 _transformation, but has not been validated on a spike-in experiment. We apply VST to the data from a recently published spike-in experiment and compare it both to a regular log_2 _analysis and a recently recommended analysis that can be applied if all raw data are available.

**Findings:**

VST provides more power to detect differentially expressed genes than a log_2 _transformation. However, the gain in power is roughly the same as utilizing the raw data from an experiment and weighting observations accordingly. VST is still advantageous when large changes in expression are anticipated, while a weighted log_2 _approach performs better for smaller changes.

**Conclusion:**

VST can be recommended for summarized Illumina data regardless of which Illumina pre-processing options have been used. However, using the raw data is still encouraged whenever possible.

## Background

Gene expression microarrays allow messenger RNA (mRNA) abundance to be quantified quickly and cost-effectively on a genome-wide scale. The production of mRNA is a key step in the process that leads from the information contained within DNA to the formation of the proteins that act within a cell. Quantifying the abundance of mRNA is therefore of interest because it provides much information regarding the state of the cell [1].

Microarrays for measuring mRNA expression make use of probes that hybridize to fluorescently-labelled sample material, where the measured level of fluorescence is used to infer the expression level of each interrogated gene. Traditionally they are constructed by attaching probes directly to a specific point on the array's surface. By contrast, the BeadArray expression platform developed by Illumina makes use of probes attached to beads that are subsequently randomly arranged on the array surface [2]. There are approximately 30 beads for each type of probe (a high degree of replication for a microarray), providing robustness against systematic spatial influences on the array.

For BeadArrays, the raw (bead-level) intensity information is stored in a proprietary format. Until recently, only summarized output (averaged values over the replicate beads on a given array) was available from Illumina's analysis software (BeadScan and BeadStudio). As such, most published studies make use of summarized Illumina data, a state that leaves the low-level (but vital) steps (e.g. image analysis, background correction, summarization) beyond the control of the data analyst.

The data from microarray experiments generally require transformation in order to facilitate simple analyses such as the confident fitting of basic linear models. Variance-stabilizing transformations are applied to microarray data in order to remove the mean-variance relationship in intensities. A log_2 _transformation is the simplest variance-stabilizing transformation commonly applied to microarray data. Other, more sophisticated approaches have been developed, such as the variance-stabilizing normalisation (VSN) method of Huber *et al. *[3] and that of Durbin *et al. *[4].

The VST method [5] is an adaptation of the VSN methodology for Illumina data, exploiting the replicate beads on the array and is defined for intensity *x *as

$$f(x)=\frac{arcsinh\left(\frac{c_{2}}{\sqrt{c_{3}}}+\frac{c_{1}x}{\sqrt{c_{3}}}\right)}{c_{1}}$$

where *c*_3 _is defined as the variance of bead types that estimate background noise and *c*_2 _and *c*_1 _respectively represent additive and multiplicative levels of error in the intensity.

Using previously published data [6], VST is found to outperform the approach of log_2 _transformation, based on the results of a mixture experiment where each sample was a pool of blood and placenta at various ratios. However, the authors commented on the then lack of a publicly available spike-in experiment, a data set that would have provided an ideal test for their method.

Coinciding with the publication of VST, Dunning *et al*. [7] published an independent account of such a spike-in experiment using customized Mouse WG-6 BeadArrays. In addition to the approximately 48,000 probes (bead types) included as standard, the content of these chips was modified to include 33 probes targeting bacterial and viral genes absent from the mouse genome. These "spikes" were added at specific concentrations on each array, and hence the relative change in expression level of a particular spike between arrays is known *a priori*. The expression levels of the remaining probes ("non-spikes") should not change between arrays. Twelve different concentrations of spike were used (1000 pM, 300 pM, 100 pM, 30 pM, 10 pM, 3 pM, 1 pM, 0.3 pM, 0.1 pM 0.03 pM, 0.01 pM and 0 pM) and each was replicated four times. Control experiments such as this have proven useful for comparing low-level analysis methods in other microarray platforms, such as Affymetrix [8].

Access to the raw bead-level data allows some of the low-level analysis steps to be explored in greater detail [7]. In particular, it was shown that the local background correction and summarization steps carried out by BeadScan and BeadStudio reduce bias and produce robust summary measurements.

The *"Background normalisation" *method (BGN) available in BeadStudio adjusts the intensities on each array by subtracting the average expression level of the negative controls (probes that have no targets in the genome being studied) in order that arrays might have comparable baselines. In the analysis of the spike-in experiment, it was shown that BGN resulted in many negative values, and also in increased variability of intensity at low expression levels when combined with the standard log_2 _transformation. Concordant with previously published observations [6], it was concluded that BGN is not desirable.

It has also been shown that, by using the variances of each bead type as inverse weights, the performance of linear models intended to detect differentially expressed (DE) genes can be improved [7]. This approach is generally only possible if bead-level data are available and a log_2 _transformation applied prior to calculating bead type averages and variances. We shall refer to this approach as a *weighted log*_2 _*analysis*. Other advantages of having access to data at the bead-level were also shown. Naturally such data allow for detailed quality control and also for greater flexibility in the choice of statistical model.

In this paper, we apply VST to data from the spike-in experiment. This offers further validation of the VST method, not only because the estimation of differential expression can be objectively assessed, but also because the microarray used is different: the mixture data used to validate VST was from a HumanRef-8 BeadArray with some 22,000 probes, rather than the 48,000 MouseWG-6 BeadArray used in the spike experiment. By design, BeadArrays with 48,000 probes tend to have many more probes at low intensity [7] than the HumanRef-8 BeadArray that only contains probes taken from a curated database. Since 48,000 probe BeadArrays are more widely used, it is important to confirm that VST can be applied to these higher density arrays with no impairment due to the different distribution of intensities. Additionally, we will investigate whether VST can reduce some of the problems encountered when applying a standard log_2 _transformation after BGN.

## Methods

The bead-level data for the spike-in experiment were read by *beadarray *[9] (version 1.7.11) using the default background subtraction method. These bead intensities were then filtered using a 3 median absolute deviation cut-off to remove outliers. The data were summarized and transformed (VST or log_2_) as appropriate, and the arrays were then quantile normalized. The bead-level data were reprocessed using both background subtraction and background normalization, and the '*lumi' *software package [5] (version 1.5.17) used to apply either a VST (with the default settings) or a log_2 _transformation (with an offset added if necessary to avoid negative values).

The linear model and subsequent analyses used to find DE genes between arrays with different spike concentrations have been previously described [7]. We obtained log-odds scores quantifying the evidence for differential expression for both the spike and non-spike probes. The 12 spike concentrations allow for construction of 6 independent contrasts. We considered two sets: one where neighbouring concentrations are compared to provide the greatest challenge for differentiation (1^st ^concentration vs 2^nd^, 3^rd ^concentration vs 4^th ^etc.) and one where a range of effect sizes would be observed by contrasting pairs symmetric about the middle concentrations (1^st ^concentration vs 12^th^, 2^nd ^concentration vs 11^th ^etc.). Finally, a series of smaller models were fitted, where only the 8 (of the 48) arrays featuring in the contrast of interest (4 arrays for each concentration) were considered.

ROC curves were also plotted, but not found to be informative as the spikes were consistently selected as DE, with very few false positives (data not shown). All data and scripts used in the analysis are available as supplementary material [10].

## Results

**Figure 1:**
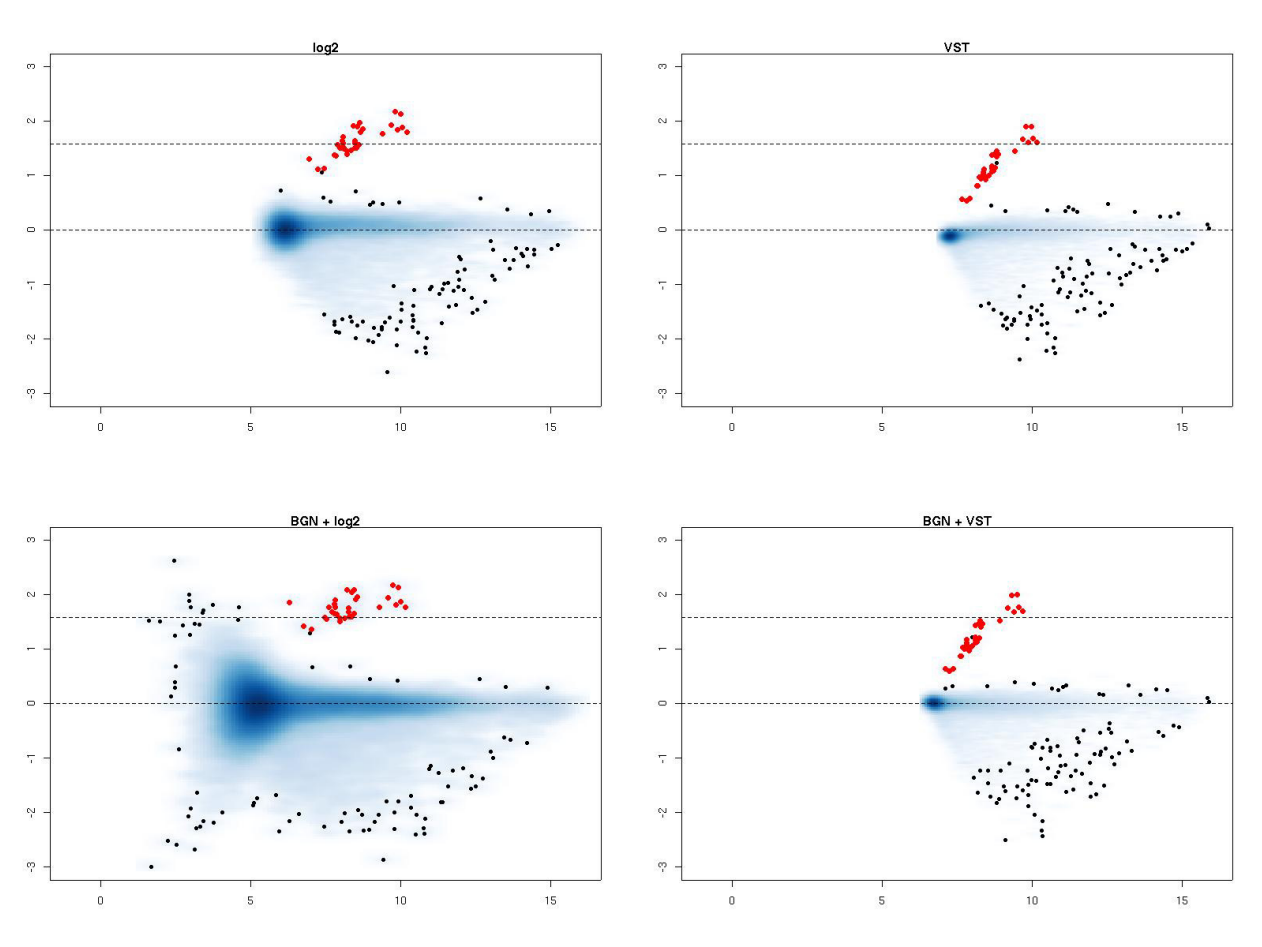
MA-plots show, for two arrays, the average log intensity (x-axis) plotted against the log-ratio of intensities (y-axis). Here, we show the MA-plots for an array with spikes at concentration 3 pM against spikes at concentration 1 pM. In the top row, the arrays were transformed with a log_2 _transformation or VST. In the bottom row, the arrays were background normalized before transformation. In all plots, red dots mark the values for the spike probes and the dotted lines indicate the predicted log fold-change of spikes (1.73) and non-spikes (0) respectively.

Figure 1 shows MA-plots comparing arrays with spikes at 3 pM and 1 pM. When BGN is not used, VST reduces the range of observed log-ratios for the probes we expect not to change. In the absence of BGN both the log_2 _transformation and VST separate the spikes well from the non-spikes, but the log-fold changes achieved from the log_2 _transformation exhibit less bias.

Applying the transformations after BGN, we see that the MA-plot for VST is little changed. By contrast, the combination of BGN and log_2 _transformation is to be avoided, with much-reduced ability to separate out the spikes from the non-spikes by considering the log_2_-ratio, as we have previously noted [7].

**Figure 2:**
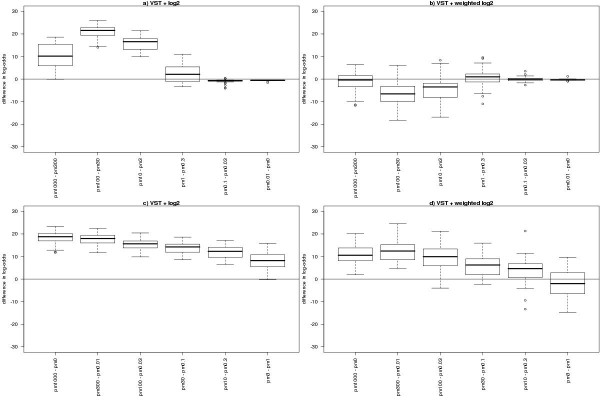
Comparison of spike log-odds obtained for a particular contrast in the linear model fitted to the entire spike-in experiment of 48 arrays. On the left we show the difference between the log-odds obtained after VST and the log-odds obtained after a log_2 _transformation. On the right, we show the difference between VST and a linear model incorporating log_2 _variances as weights (see [7]). In the top panels, we show six independent contrasts with the closest spike concentrations. The bottom panel shows six independent contrasts from the same linear model, but chosen to provide a range in anticipated log-ratios (the finer differences being to the right of the panel). In all cases, a positive value indicates greater log-odds obtained (i.e. more evidence for differential expression) after VST.

We fitted three linear models to the entire spike-in experiment: one using VST, one using a log_2 _transformation, and the *weighted log*_2 _*analysis*. For two of the linear models at a time, Figure 2 displays the differences in log-odds calculated for six contrasts. VST is seen to lead to a more powerful test than a standard log_2 _transformation, producing higher log-odds values for the spikes (Figure 2a/2c). At the same time, values for the non-spikes were not appreciably altered (data not shown). The difference between VST and log_2 _is seen to decrease as the spike concentrations get closer together (Figure 2c).

When comparing VST to a *weighted log*_2 _*analysis *(Figure 2b/2d), VST is seen to be more powerful for detecting differential expression for large differences, but the *weighted log*_2 _*analysis *outperforms VST for finer comparisons (such as 100 pM vs 30 pM and 3 pM vs 1 pM).

**Figure 3:**
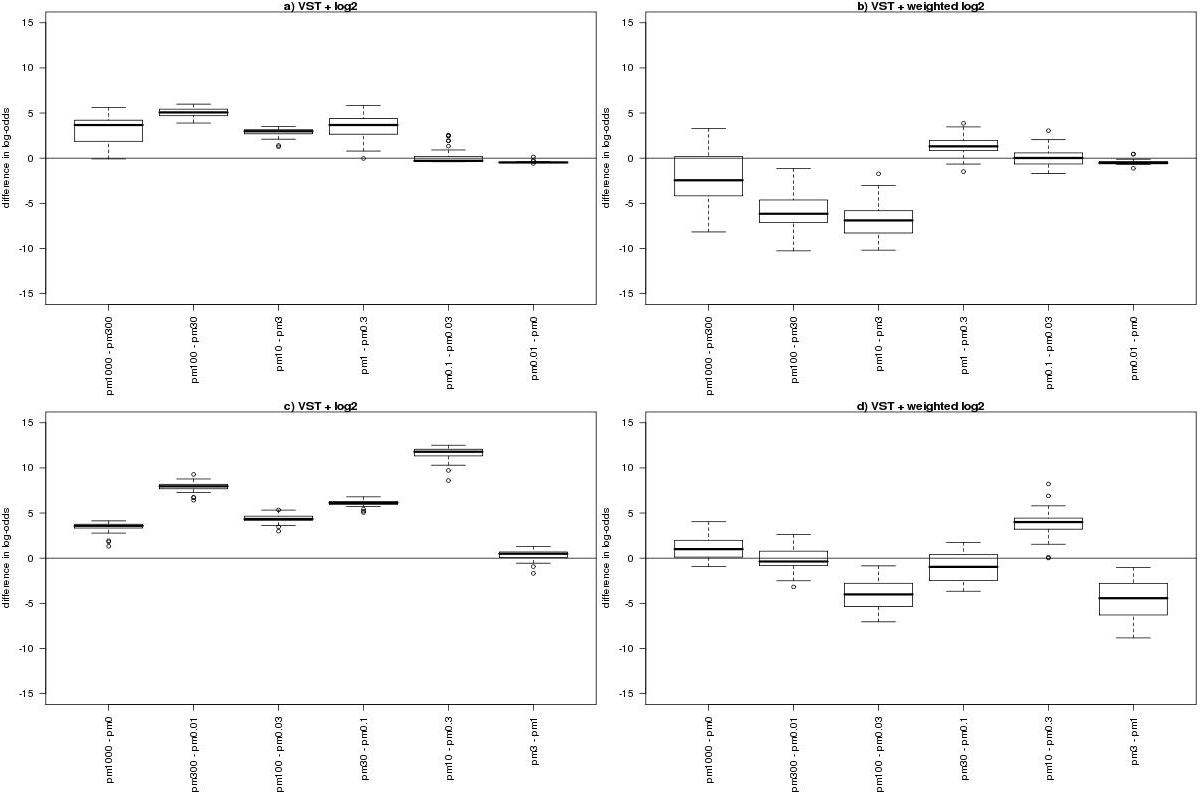
Comparison of spike log-odds obtained for a particular contrast in the linear model fitted to the 8 arrays involved in that contrast. On the left we show the difference between the log-odds obtained after VST and the log-odds obtained after a log_2 _transformation. On the right, we show the difference between VST and a linear model incorporating log_2 _variances as weights (see [7]). In the top panels, we show six independent contrasts with the closest spike concentrations. The bottom panel shows six independent contrasts chosen to provide a range in anticipated log-ratios (the finer differences being to the right of the panel). In all cases, a positive value indicates greater log-odds obtained (i.e. more evidence for differential expression) after VST.

When the models are fitted to only the arrays involved in the contrast of interest (Figure 3), the same broad results are seen. The *weighted log*_2 _*analysis*, however, begins to show more sensitivity than VST even at quite extreme comparisons (e.g. 100 pM vs 0.03 pM).

## Discussion

In agreement with the original investigation into VST, we find that VST offers improvements over a standard log_2 _analysis. Thus, users with only the summarized output from BeadStudio will find this method beneficial. In particular, VST can cope with data that have been background normalized (BGN is implemented as the *"subtract background" *option in recent versions of BeadStudio). This should not be confused with local background subtraction that has already been applied to bead-level data prior to summarization.

Using a published spike-in experiment we are also able to show that VST offers greater ability to detect DE genes compared to a log_2 _transformation. This improvement was seen to diminish as the spike concentrations being compared become closer. At the same time, a *weighted log*_2 _*analysis *had more power than VST for finer concentration differences.

In our initial analysis of the spike-in experiment, we used all 48 arrays in the linear model. The size of such an experiment may not be typical for some researchers and therefore we repeated the analysis using fewer arrays. In this smaller experiment, VST was seen to have marginally improved log-odds over a regular log_2 _analysis. Under these conditions the *weighted log*_2 _*analysis *was seen to improve the detection of DE genes in most cases, especially when comparing arrays with similar spike concentrations. We note that a *weighted log*_2 _*analysis *is compromised without access to bead-level data. It would be beneficial if Illumina's software had the option to work with data on the log_2 _scale when creating summarized data.

In summary, we have shown that the VST method does indeed perform well, and can be applied to the popular 48,000 probe BeadArrays. However, there are still benefits to having access to the raw data.

## Authors' contributions

MJD performed the analysis under the supervision of MER and AGL. NLBM provided expertise on the comparison of arrays. The study was conceived out of a discussion between all of the authors. MJD, MER and AGL drafted the manuscript. All authors read, revised and approved the final manuscript.
